# Characterization of Anti-Citrinin Specific ScFvs Selected from Non-Immunized Mouse Splenocytes by Eukaryotic Ribosome Display

**DOI:** 10.1371/journal.pone.0131482

**Published:** 2015-07-01

**Authors:** Haiwei Cheng, Yifei Chen, Yi Yang, Xueqiu Chen, Xiaolu Guo, Aifang Du

**Affiliations:** Institute of Preventive Veterinary Medicine & Zhejiang Provincial Key Laboratory of Preventive Veterinary Medicine, College of Animal Sciences, Zhejiang University, Hangzhou, China; Imperial College London, UNITED KINGDOM

## Abstract

Single chain variable fragments (scFvs) against citrinin (CIT) were selected from a scFv library constructed from the splenocytes of non-immunized mice by an improved eukaryotic ribosome display technology in this study. Bovine serum albumin (BSA)/ CIT-BSA and ovalbumin (OVA)/ CIT-OVA were used as the antigens to select specific anti-CIT scFvs. Eukaryotic *in situ* RT-PCR method was used to recover the selected mRNA after every affinity selection. After six rounds of ribosome display, expression vector pTIG-TRX carrying specific scFv DNAs were constructed and transformed into *Escherichia coli* BL21 (DE3) for protein expression. Thirteen positive clones were selected out of which three (designated 23, 68 and 109) showed high binding activity and specificity to CIT by indirect ELISA, while no clone showed binding activity with carrier proteins. The three scFvs showed high specificity to CIT and the cross reactivity with other mycotoxins was below 0.01% as determined by indirect competitive ELISA. These specific scFvs offer a potential novel immunoassay method for CIT residues. This study confirmed the effectiveness of the improved eukaryotic ribosome display system and could be used as a reference for the selection of scFvs specific to other small molecules using ribosome display.

## Introduction

**Fig 1:**
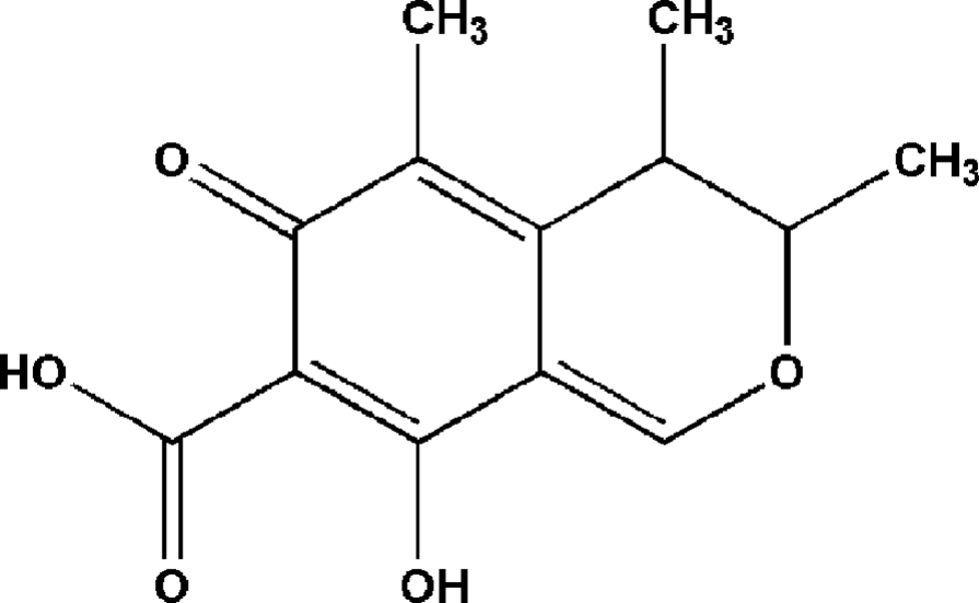
Chemical structure of citrinin (CIT).

CIT (Fig 1) is a hepato-nephrotoxic fungal metabolite produced by the genera *Penicillium* [1], *Aspergillus* [2] and *Monascus* [3], originally isolated in 1931. It was once used clinically as an experimental antibiotic [4]. However, subsequent research indicated that CIT often occurs in naturally contaminated commodities, such as corn, barley, wheat, apple and other food products [5–8], behaving as a nephrotoxin in animals [9], damaging proximal tubules of the kidney [10], and was implicated as a potential causative agent in human endemic Balkan nephropathy. Nowadays, it is known to be one of the main mycotoxins that lead to several serious health problems [11].

Currently, different methods have been reported for the analysis of CIT residues, such as thin-layer chromatography [12], high-performance liquid chromatography [13], liquid chromatography with mass spectrometry [14] and gas chromatography with mass spectrometry [15]. However, these methods require well-equipped laboratory, trained personnels, high capital expenditure and time-consuming sample preparation steps. Therefore, there is a great demand for more economical, rapid, and portable methods for the detection of CIT residues.

Immunoassays such as enzyme linked-immunosorbent assay (ELISA) have been exploited [16]. However, for many years this application has been relying heavily on the utilization of antibodies, of which were generated through animals immunization. As a hapten, the preparation of anti-CIT antibodies is time consuming and technically demanding. The recombinant antibody display technology poses certain advantages over the hybridoma technology, while phage display is now widely used for the selection of antibody fragments [17].

Recently, as a more effective display system, ribosome display has been reported [18–20]. It is an *in vitro* display method that links phenotype directly to genotype by non-covalent ternary polypeptide-ribosome-mRNA complexes [21, 22]. Compared to other display systems such as phage display, ribosome display offers unique superiority in antibody selection. For instance, ribosome display avoids transformation limits, enlarges the capacity of the library, enriches the diversity of target molecules [23, 24], introduces random mutations by PCR, encompasses powerful capabilities in molecular evolution and affinity maturation, and allows the expression of toxic and chemically modified proteins [25–27]. Thus far, ribosome display technology has been used to select scFvs in various fields [28–37], while work on the selection of scFvs against CIT by ribosome display has been limiting since it was first reported in 1994 [18].

In this study, an improved eukaryotic ribosome display combined with nested PCR was used to select anti-CIT scFvs from a scFv library constructed from the splenocytes of non-immunized mice, aiming to develop a novel immunoassay method for CIT residues.

## Materials and Methods

### Materials

Citrinin (CIT), 1-ethyl-3-(3-dimethylaminopropyl) carbodiimide hydrochloride (EDC), *N*-hydroxysuccinimide (NHS) and 2-(morpholino) ethanesulfonic acid (MES) were purchased from Sigma (St. Louis, MO, USA). TRIZOL and Mouse anti-His tag monoclonal antibody were purchased from Invitrogen (USA). ReverTra Ace-α- was purchased from Toyobo (Japan). The pMD18-T vector and primerSTAR MAX DNA polymerase were purchased from TaKaRa Biotechnology Ltd. (Dalian, China). TNT Quick Coupled Transcription/Translation Systems, Wizard SV Gel and PCR Clean-up System, and RNase-free DNaseІ were purchased from Promega (USA). The pTIG-TRX vector was provided by Beijing Institute of Biotechnology (Beijing, China). A HisTrap HP metal affinity chromatography system was purchased from GE Healthcare (USA). HRP-labeled goat anti-mouse IgG (H+L) was purchased from BioWorld (USA). The CIT-protein conjugate was determined using UV-2550 spectrophotometer (Shimadzu, Japan) and TripleTOF 5600+ (AB SCIEX, USA).

### Synthesis and Identification of CIT-Protein Conjugates

CIT was coupled covalently to the carrier protein bovine serum albumin (BSA) *via* amide bonds, using the active ester method using 1-ethyl-3-(3-dimethylaminopropyl) carbodiimide hydrochloride (EDC) and *N*-hydroxysuccinimide (NHS) as dehydrating agent. The coupling ratio of hapten to protein was 5:1 to 20:1. For the synthesis of conjugate, 2 mg CIT in 2 mL MES Buffered Saline (0.1 M MES [2-(morpholino) ethanesulfonic acid], 0.5 M NaCl, pH 6.0) was mixed with 0.4 mg EDC and 0.6 mg NHS. After reacting for 15 minutes at room temperature 1.4 μL 2-mercaptoethanol was added to the MES Buffer to quench the EDC, followed by the addition of 1 mL protein solution (10 mg/mL BSA in 0.1 M phosphate buffered saline (PBS)). The solution was mixed well and then allowed to react for 2 hours at room temperature. Finally, the conjugates were dialyzed with 0.01 M phosphate buffered saline (PBS) at 4°C for 2 days. Fresh PBS was replaced every eight hours. The conjugate was lyophilized and stored until use. The CIT-BSA conjugate after preparation was primarily identified by sodium dodecyl sulfate polyacrylamide gel electrophoresis (SDS-PAGE) and UV-2550 spectrophotometer. The molar ratio of CIT-BSA in the conjugate was determined by MALDI-TOF-MS. The CIT-OVA conjugate was synthesized using the same method.

### Construction of the ScFv Library

Total RNA was isolated from splenocytes of non-immunized of mouse with TRIZOL according to the manufacturer’s instructions (Invitrogen, USA). cDNA was amplified by RT-PCR using ReverTra Ace-α- (Toyobo, Japan). The primer pairs V_H_R (5’-SAGGTSCASCTCGAGSAGTCTGG-3’) [30] and V_H_F (5’-TGAGGAGACGGTGACCGTGGTCCCTTGGCCCC-3’) [38] were used to amplify the V_H_ chain. The primer pairs V_L_R (5’-GAYATTGTGYTRACACAGTC-3’) and V_L_F (5’-ACGTTTKGATTTCCAGCTTGG-3’) were used to amplify the V_L_ chain. A 85-bp DNA linker containing a sequence encoding (Gly_4_Ser) _3_ was amplified with primers Linker-R (5’- CCACGGTCACCGTCTCCTCAGGCGGCGGCGGCTC -3’) and Linker-F (5’- GACTGTGTYARCACAATRTCGGAGCCTCC -3’). After gel purification with Wizard SV GEL kit (Promega, USA), the V_H_, V_L_ and the linker fragments were assembled into full-length scFv by splicing overlap extension PCR (SOE-PCR). Briefly, 15 ng of V_H_ DNA, 25 ng of linker DNA and 15 ng of V_L_ DNA were mixed with 25 μL of PCR mixture without primers and run for 10 cycles (30 s at 95°C, 120 s at 68°C), after which primers V_H_R and V_L_F were added. The mixture was amplified for 25 cycles (30 s at 95°C, 30 s at 55°C, 90 s at 72°C). The purified scFv library was cloned into pMD18-T vector (TaKaRa, Japan) and transformed into calcium-competent *E*. *coli* TOP10 cells. Eighteen randomly chosen clones were sequenced.

Mice used as experimental animals, were treated in strict accordance with the recommendations in the Guide for the regulation for the Administration of Affairs concerning Experimental Animal of the People’s Republic of China. Animal experiments were approved by Zhejiang University Experimental Animal Ethics Committee (Permit Number: ZJU201308-1-10-072). Mice were humanely euthanized under sodium pentobarbital anesthesia, and all efforts were made to minimize suffering.

### Generation of the Ribosome Display Library

To construct a complete ribosome display library, the T7 promoter, ribosome binding sites and a spacer sequence were added to the scFv library. The T7R primer (5’-GCAGCTAATACGACTCACTATAGGAAGAACAGA **CCACCATG**SAGGTSCASCTCGAGSAGTCTGG-3’) harbored a T7 promoter (underlined), a ribosome binding site and initiation codon ATG (bold) for transcription and translation *in vitro*. The constant region of κ chain (Cκ) was used as the spacer sequence and the primer pairs CκR (5’-CCAAGCTGGAAATCMAAACGTGCTGATGCTGCACC-3’) and CκF(5’- CACTCATTCCTGTTGAAGCTCTTG-3’) [20] were used to amplify the Cκ chain. The T7 promoter, ribosome binding sites, scFv library and the Cκ fragment were assembled into the ribosome display library by SOE-PCR. The purified library was cloned into pMD18-T vector (TaKaRa, Japan) and transformed into calcium-competent *E*. *coli* TOP10 cells. Fifteen randomly chosen clones were sequenced.

### ARM Ribosome Display

Eukaryotic ribosome display was performed in a coupled transcription/translation system (Promega, USA) as described [20, 39, 40] with some modifications. Briefly, 40 μL TNT Quick Master Mix, 1 μL 1 mM Methionine, 0.5 μL 100 mM magnesium acetate and 1 μL T7 TNT PCR Enhancer were mixed in a RNase-free tube with 1~2 μg purified ribosome display library. After incubation at 30°C for 60 mins, 50 units RNase-free DNaseІ, 6 μL 10 × DNase I digestion buffer (400 mM Tris/HCl, pH 8.0, 100 mM MgSO_4_, 10 mM CaCl_2_) were added and the total volume was adjusted to 70 μL with ice-cold nuclease-free water. After incubating at 30°C for an additional 20 mins, the reaction was arrested by cooling on ice and the product was diluted with 210 μL of ice-cold PBS (containing 5 mM magnesium acetate and 2.5 mg/mL heparin) immediately [37, 41]. Untranslated mixture (translated for 0 min) was used as a negative control. The antibody-ribosome-mRNA (ARM) ternary complexes were then used immediately for affinity selection.

### Affinity Selection and *in situ* RT-PCR Recovery

Affinity selection was performed as described [20, 40, 41] with some modifications. Microtiter plates were coated at 4 ^o^C overnight with 50 μL of CIT-BSA solution (0.2 mg/mL in carbonate buffer solution) or BSA solution (0.2 mg/mL in carbonate buffer solution). The coated plates were washed with PBST (PBS with 0.05% (v/v) Tween 20) and blocked with sterilized 5% (w/v) skim milk in PBS for 2 h at room temperature. The plates were then washed five times with ice-cold washing buffer PBSTM (PBS with 0.05% (v/v) Tween 20 and 5 mM magnesium acetate) and incubated on ice for 20 mins. The prepared TNT translation mixture containing the ARM complexes was first added to the prepared BSA-coated microtiter well for pre-binding and the plates were incubated at 4°C for 1 h with gentle shaking. Supernatant containing the rest of the ARM complexes was transferred into the prepared CIT-BSA-coated microtiter well and incubated at 4°C for 2 h. Following three washes with cold washing buffer and two quick washes with ice-cold nuclease-free water, *in situ* RT-PCR recovery was performed in the wells using primer D2 (5’-CGTGAGGGTGCTGCTCATG-3’) for the first cycle. In short, 11 μL RNase-free H_2_O and 1 μL primer D2 (10 μM) were added into the wells. After incubating at 65°C for 5 mins and putting on ice for at least 30s, the following reagents were added: 4 μL 5×RT buffer, 2 μL dNTP mixture (10 mM each), 10 units RNase inhibitor and 1 μL ReverTra ACE. The wells were incubated at 30°C for 10 mins, 42°C for 30 mins, 85°C for 5 mins and 4°C for 5 mins in that order. The obtained cDNA was amplified using primers T7R and D2. After purification with Wizard SV Gel and PCR Clean-up System, the selected library could be cloned into the T-cloning vector for either sequencing or subsequent cycles.

The second round of *in vitro* selection was performed with almost the same conditions, except that the microtiter plates were coated with the CIT-OVA conjugate and different primers were used. CIT-OVA and CIT-BSA were used to coat the microtiter plates in turn to isolate specific anti-CIT scFv. Primers D3 (5’-GGGGTAGAAGTTGTTCAAGAAG-3’) and D4 (5’-CTGGATGGTGGGAAGATGG-3’) were used in the second and third round, respectively.

### Cloning and *E*. *coli* Expression

The selected anti-CIT scFvs were cloned into pMD18-T vector (TaKaRa, Japan) after the sixth round of selection and transformed into calcium-competent *E*. *coli* TOP10 cells. Based on the sequencing results, the clones in right reading frame without stop codon were chosen for prokaryotic expression.

The chosen scFvs were amplified with the primer pairs VH-*EcoR І* (5’-GAATTCTAAATGSAGGTSCASCTCGAGSAGTCTGG-3’) and VL-*Hind Ⅲ* (5’-AAGCTTACGTTTKGATTTCCAGCTTGG-3’) (the restriction sites *EcoR І* and *Hind Ⅲ* are underlined, respectively). The PCR reaction was performed with primerSTAR MAX DNA polymerase (TaKaRa, Japan) for 30 cycles (98°C for 10 s, 55°C for 10 s, 72°C for 30 s). The amplified scFv DNA and vector pTIG-TRX [42] were digested with *EcoR І* and *Hind Ⅲ* and purified using the Wizard SV Gel and PCR Clean-up System. Ligations of prepared insert DNA and pTIG-TRX vector were carried out using T4 DNA ligase (TaKaRa, Japan). Ligated products were transformed into *Escherichia coli* BL21 (DE3) and soluble proteins were expressed from each clone. Briefly, single colony was cultured in 50 mL of LB medium with 100 μg/mL ampicillin at 37°C /250 rpm until they reached an OD_600_ of 0.7. Isopropyl b-d-thiogalactopyranoside (IPTG) was added to a final concentration of 1 mM, and induced at 25°C /130 rpm for 5~7 h. Each culture was then centrifuged at 8,000 rpm for 10 mins. Cell pellet was resuspended in 7 mL of 50 mM PBS (pH 7.4) and the cells were ruptured by sonication followed by centrifugation at 8,000 rpm for 10 mins. Both periplasmic extracts and precipitates were collected and tested for the status of expressed scFvs by SDS-PAGE.

### Western Blot Analysis of ScFvs

Supernatants of the expressed scFvs were analyzed by Western blotting using the mouse anti-His tag antibody as the pTIG-TRX vector harbors a His-tag. The supernatants were subjected to SDS-PAGE on 12% polyacrylamide gel. Pre-stained SDS-PAGE standards (Fermentas, USA) were used to calibrate protein molecular size. The gel was transferred onto a nitrocellulose membrane using semidry electroblotter (Bio-Rad, USA) after SDS-PAGE. The membrane was blocked for 1 h at 37°C with blocking solution (5% (w/v) skim milk in TBST) and then incubated for 1 h at 37°C with the mouse anti-His tag antibody, followed by incubation for 1 h at 37°C with HRP-labeled goat anti-mouse IgG. 4-CN (4-chloro-1-naphthol, Sigma) was used as the peroxidase substrate.

### ELISA Analysis of the Binding Activity of ScFvs to CIT

To screen for specific anti-CIT scFvs, the expressed soluble scFvs of each clone was analyzed by indirect ELISA. Microtiter plates were coated with 100 μL of CIT-BSA solution (0.2 mg/mL in 0.05 M sodium carbonate-bicarbonate buffer, pH 9.6) or BSA solution (0.2 mg/mL in 0.05 M sodium carbonate-bicarbonate buffer, pH 9.6) as control overnight at 4°C. The plates were washed five times with PBST (10 mM PBS containing 0.05% Tween 20, pH 7.4) and blocked for 1 h at 37°C with 5% (w/v) skim milk in PBS. 100 μL of periplasmic extracts were added to the antigen-coated wells and incubated for 2 h at 37°C after the wells were washed. The mouse anti-His tag antibody and HRP-labeled goat anti-mouse IgG(H+L) were used as primary and secondary antibody in tandem. Signals were developed with OPD-detection-solution and outputs read at OD_492_ nm.

### Specificity and Affinity Analysis of ScFvs

Indirect competitive ELISA was performed to assess the specificity and affinity of the scFvs [43]. Different concentrations of free CIT were prepared with PBST containing 10% methanol (from 0.001 μg/mL—100 μg/mL) and the scFv solution was preincubated with different concentrations of free CIT, respectively. The scFv-CIT mix was added to microtiter wells coated with 0.2 mg/mL CIT-BSA and incubated for 2 h at 37°C. Bound scFvs were detected as described above while unbound antibodies were washed with PBST. The binding rate (B/B0) was calculated according to the following equation: B/B0 (%) = (OD_492 nm_ at certain concentration of free CIT/OD_492 nm_ at zero concentration of free CIT) × 100. Apparent affinities were determined as the reciprocal of molar concentration of the CIT required to inhibit 50% maximal binding in the competitive ELISA (molarity of the IC_50_ of CIT).This is a close approximation to the true affinity [43] and permitted the ranking of the binding activities.

Patulin, Aflatoxin B1, Fumonisin B1 and Ochratoxin A (Sigma, USA) were used to evaluate the cross-reactivity (CR) of the scFvs and the CR values were calculated according to the following equation: CR (%) = (IC_50_ of CIT/IC_50_ of other mycotoxins) × 100.

### Expression and Purification of the Anti-CIT ScFv

Positive clones binding to CIT were grown in 500 mL cultures and the expressed anti-CIT scFv was extracted as described previously. Soluble scFv was purified by HisTrap HP metal affinity chromatography (GE Healthcare, USA) following the manufacturer’s instructions. Eluted scFv was tested by Western blotting.

## Results and Discussion

### Identification of the CIT-BSA Conjugate

**Fig 2:**
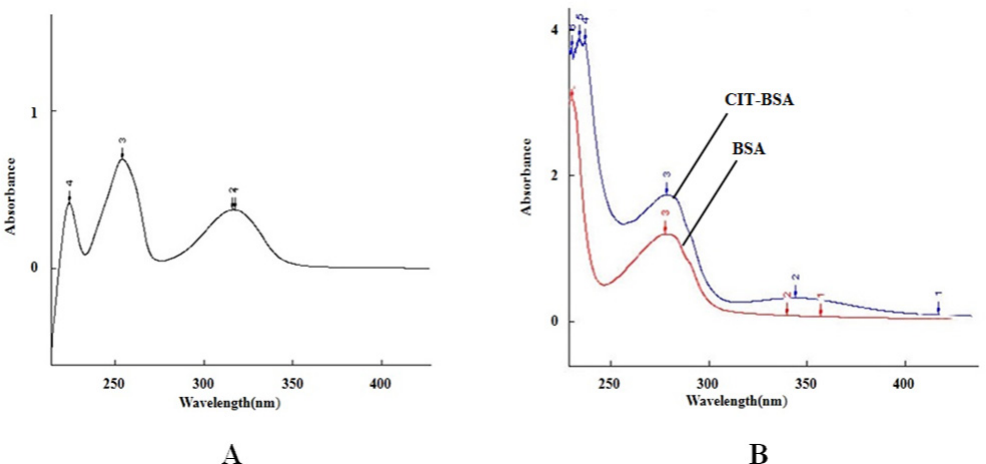
Ultra-violet spectrograms of CIT, BSA and CIT-BSA conjugate. A. Ultra-violet spectrograms of CIT.The ultraviolet absorption peak of CIT was at 319 nm. B. Ultra-violet spectrograms of BSA and CIT-BSA conjugate. The ultraviolet absorption peaks of CIT-BSA conjugate were at 280 nm and 330nm, while that of BSA was at 280 nm.

**Fig 3:**
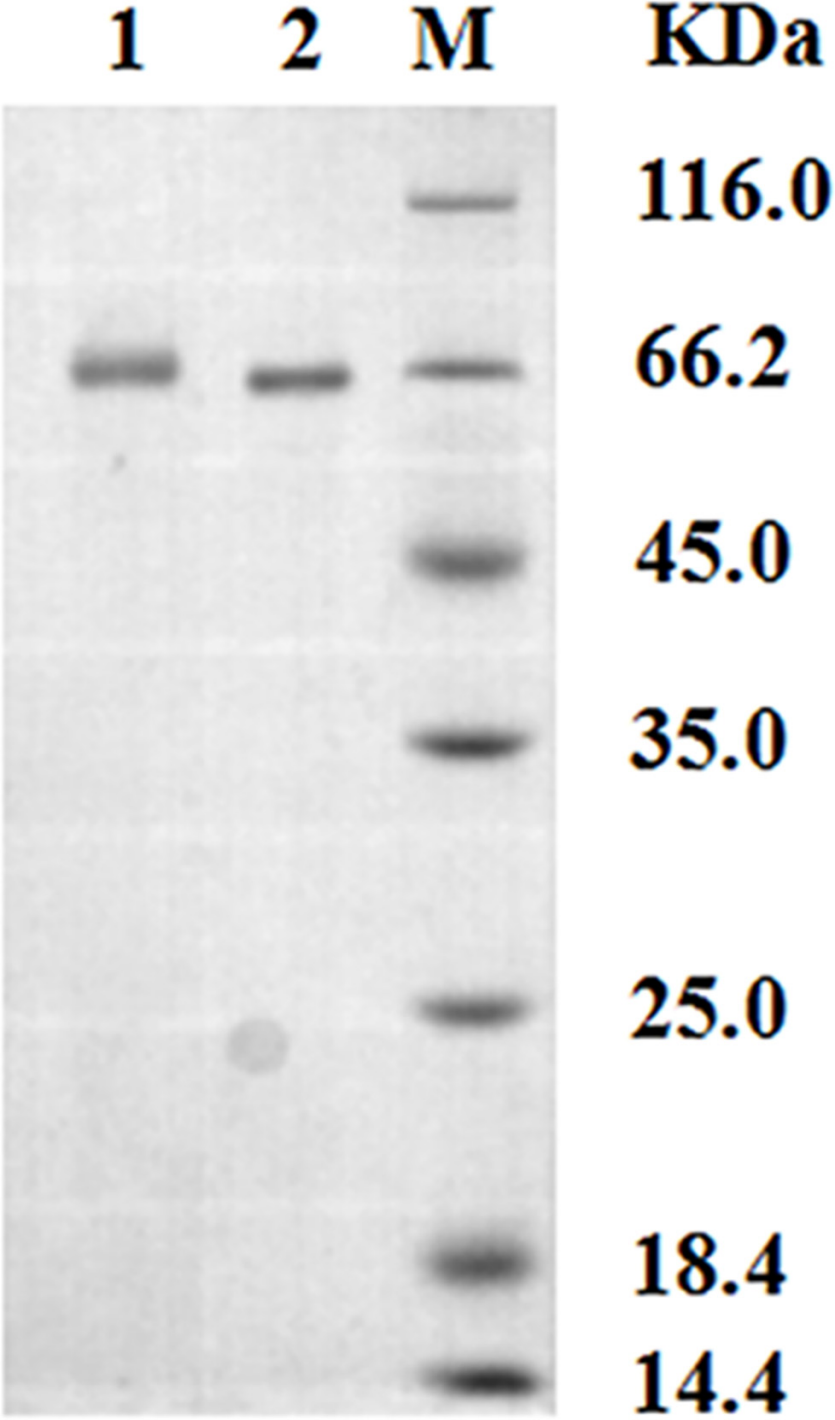
SDS-PAGE analysis of BSA and CIT-BSA conjugate. Lane 1: CIT-BSA conjugate; Lane 2: BSA; Lane M: protein standards.

**Fig 4:**
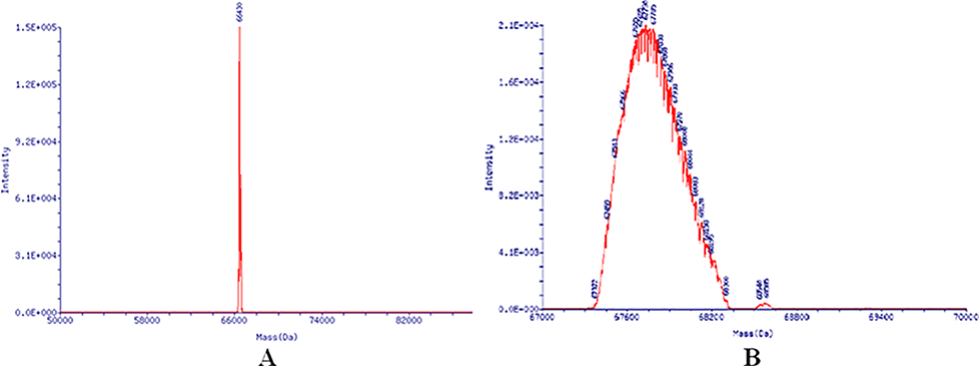
Mass spectrograms of BSA and CIT-BSA conjugate. A. Mass spectrograms of BSA. The molecular weight (M_W_) of BSA as determined by MS is 66,430 Da. B. Mass spectrograms of CIT-BSA conjugate. The molecular weight (M_W_) of CIT-BSA conjugate as determined by MS is 67,730 Da.

UV spectra and SDS-PAGE were used to identify the CIT-BSA conjugate. The UV spectra of CIT, BSA and CIT-BSA are shown in Fig 2 The ultraviolet absorption peaks of CIT-BSA conjugate were at 280 nm and 330nm, while that of CIT and BSA were at 319 nm and 280 nm, respectively. SDS-PAGE analysis of BSA and CIT-BSA is in Fig 3, showed that the molecular weight (M_W_) of CIT-BSA is higher than that of BSA. The molecular weights (M_W_) of CIT-BSA and BSA as determined by MS are 67,730 Da and 66,430 Da, respectively, as shown in Fig 4 and their coupling ratio was about 5:1. Based on the results above, it could be inferred that the CIT-BSA conjugate has been prepared and the conjugate could be used as antigen for subsequent selection.

### Generation of the ScFv Library

**Fig 5:**
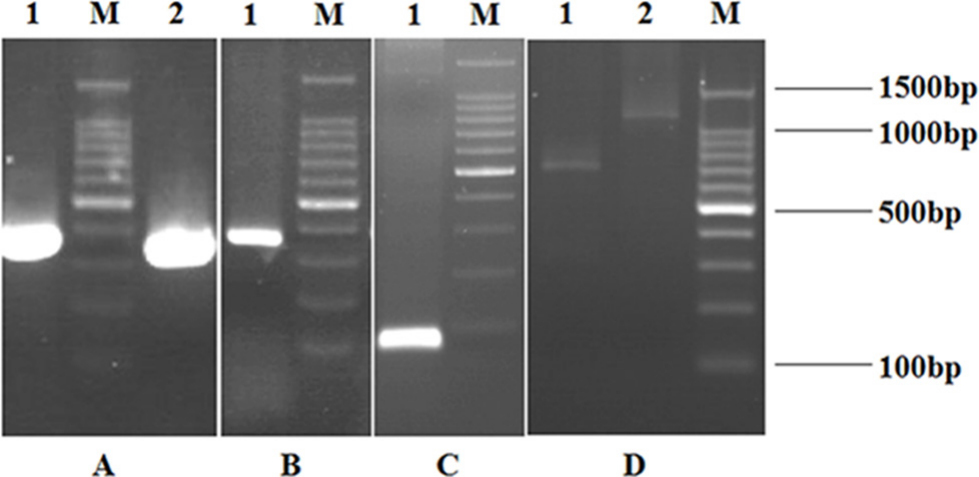
Agarose gel electrophoresis of the amplified DNA fragments of VH, VL, Cκ, GS Linker and assembled scFv library, assembled ribosome display library. A: The amplified DNA fragments of VH and VL. Lane 1: The fragment of VH; Lane 2: The fragment of VL; Lane M: 100bp DNA ladder marker. B: The amplified DNA fragment of Cκ. Lane 1: The fragment of Cκ; Lane M: 100bp DNA ladder marker. C: The amplified DNA fragment of GS Linker. Lane 1: The fragment of GS Linker; Lane M: 100bp DNA ladder marker. D: The amplified DNA fragments of assembled scFv library and ribosome display library. Lane 1: The fragment of assembled scFv library; Lane 2: The fragment of assembled ribosome display library; Lane M: 100bp DNA ladder marker.

The V_H_ and V_L_ fragments of the expected size were amplified (Fig 5). The (Gly_4_Ser) _3_-linker fragment was synthesized with the two ends complementary to the downstream sequence of the V_H_ fragments and the upstream sequence of the V_L_ fragments, respectively (Fig 5). The scFv library, used to construct the ribosome display library, was assembled with the V_H_, V_L_, and the (Gly_4_Ser) _3_-linker by SOE-PCR (Fig 5).

**Fig 6:**
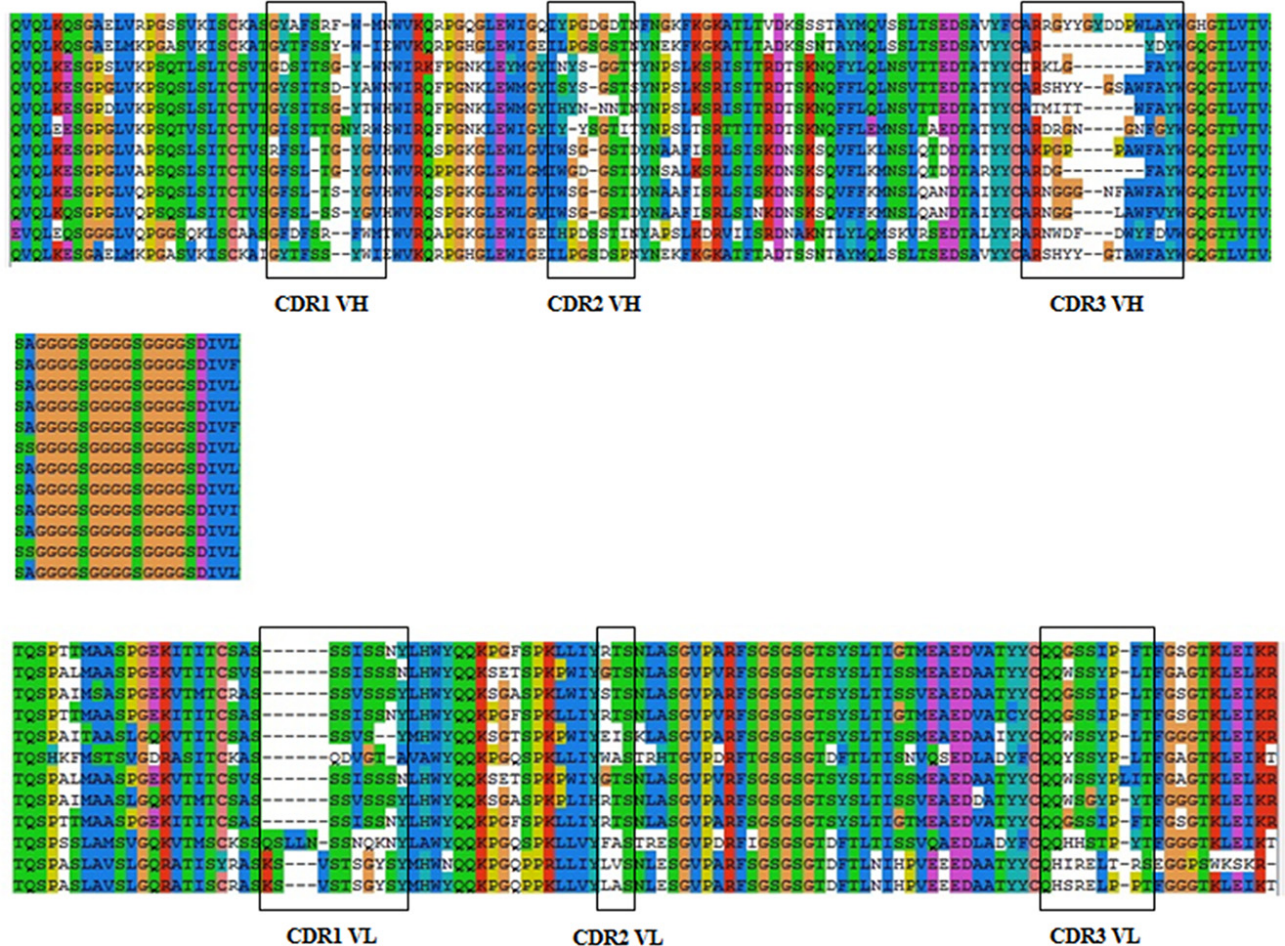
Alignment of the derived amino acids sequences of the randomly chosen scFvs and complementary determining regions (CDRs). FRs and CDRs are determined by the V-BASE DNAPLOT software.

Eighteen randomly chosen clones of the scFv library were sequenced to evaluate the integrity and diversity of the library. Twelve sequences were found to be in the right reading frame with no stop codon. The derived amino acid sequences and complementary determining regions (CDRs) are shown in Fig 6 The framework regions (FRs) and CDRs were determined by the V-BASE DNAPLOT software. The length of CDRs was variable. The length of CDR1 VH ranged from 9 to 11 amino acid residues, with an average length of 10 residues, CDR2 VH with an average length of 7 residues, CDR3 VH with an average length of 12 residues, CDR1 VL with an average length of 8 residues, while 3 amino acid residues were found in CDR2 VL and 9 amino acid residues were found in CDR3 VL. The diversity of the CDRs amino acid sequences indicated that the constructed scFv library could be used for the selection of specific anti-CIT scFv.

### Generation of the Ribosome Display Library

To link the ribosome and provide sufficient space for the displayed protein to fold on the ribosome surface, a fragment of at least 23–30 amino acids in length is required at the C-terminus of the scFv library [44]. A variety of fragments have been used, such as the constant region of immunoglobulin (Ig) κ chain, gene Ⅲ of filamentous phage M13, the CH_3_ domain of human IgM, streptavidin and glutathione-S-transferase [45].

The constant region of immunoglobulin (Ig) κ chain was used as the spacer domain in this study and the stop codon of the domain was deleted to ensure the ribosome stayed at the terminal end of the mRNA after translation was completed and stable ARM ternary complexes were generated. Another important function of Cκ was to provide a sequence for the design of primers for *in situ* RT-PCR recovery after selection [20].

The Cκ fragment was amplified with the expected size and the ribosome display library was assembled with the expected size by SOE-PCR (Fig 5).

Fifteen clones of the ribosome display library were sequenced and ten were in the right reading frame, with the T7 promoter, ribosome binding sites, scFv library and the Cκ fragment with no stop codon. The assembled ribosome display library was then used for *in vitro* transcription and translation.

### Selection of ScFvs with Affinity for CIT

The ribosome display library was subjected to the TNT Quick Coupled Transcription/Translation Systems to generate the ARM ternary complexes, which could provide a eukaryotic condition for efficient translation and folding of the scFvs. Microtiter plates coated with BSA and CIT-BSA, respectively, were used for affinity selection. The whole selection process should be performed on the ice or at 4°C and 5 mM magnesium acetate was added to the washing buffer PBST to inhibit the RNase activity and generate stable ARM ternary complexes. The ARM complexes were subjected to RT-PCR to recover the selected mRNA after every round of affinity selection.

Prokaryotic ribosome disruption [19] and eukaryotic *in situ* RT-PCR methods [20] are commonly used for RT-PCR recovery. The ARM complexes are dissociated by EDTA in the prokaryotic ribosome disruption system and the released mRNA is recovered for RT-PCR, while eukaryotic *in situ* RT-PCR operates directly on the ARM complexes without disruption and purification of the mRNA. The prokaryotic ribosome disruption protocol could lead to a poor recovery from rabbit reticulocyte lysate ribosome complexes [39] and a significant portion of the mRNA remained associated with the ribosome complexes even after EDTA treatment [46].

Eukaryotic *in situ* RT-PCR combined with nested PCR were used in this study to recover the selected mRNA as this approach could simplify the recovery process and avoid loss of selected mRNA.

**Fig 7:**
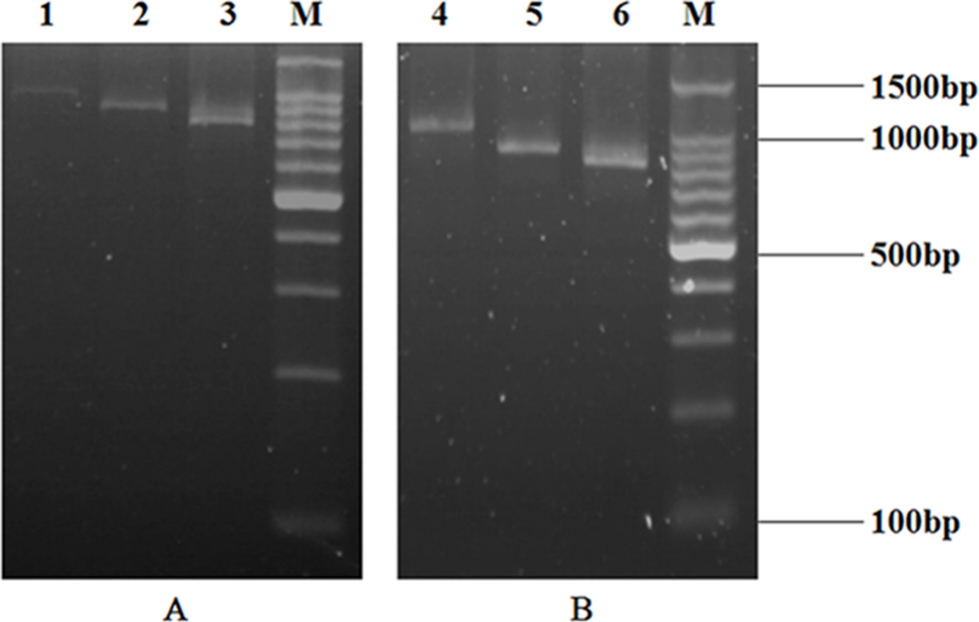
Agarose gel electrophoresis of the selected anti-CIT scFv genes over six rounds of ribosome display. After selection, the mRNA was amplified by *in situ* RT-PCR and the products were analyzed by agarose gel electrophoresis. A: The selected anti-CIT scFv genes over ribosome display. Lane 1: The selected anti-CIT scFv genes over the first round of ribosome display; Lane 2: The selected anti-CIT scFv genes over the second round of ribosome display; Lane 3: The selected anti-CIT scFv genes over the third round of ribosome display; Lane M: 100bp DNA ladder marker. B: The selected anti-CIT scFv genes over ribosome display. Lane 4: The selected anti-CIT scFv genes over the fourth round of ribosome display; Lane 5: The selected anti-CIT scFv genes over the fifth round of ribosome display; Lane 6: The selected anti-CIT scFv genes over the sixth round of ribosome display; Lane M: 100bp DNA ladder marker.

The progress of selection was monitored by estimating the enrichment of PCR products on agarose gel-electrophoresis. After the first round of selection, *in situ* RT-PCR recovery was performed in the wells carrying selected ARM complexes using the primer D2. A weak DNA band was observed (Fig 7). Six rounds of affinity selection were performed and primer D2 was used in the first and fourth rounds, primer D3 was used in the second and fifth rounds while primer D4 was used in the third and sixth rounds. Although the use of primer D2, D3, D4 in the *in situ* reverse transcription reaction would result in getting progressively shorter DNA fragments, the shortening only affects the spacer domain of the Cκ chain. A full-length library was regenerated by SOE-PCR after the third cycle [37]. The enrichment of PCR products continued during the next rounds of panning (Fig 7).

During the affinity selection, microtiter plates coated with BSA and CIT-BSA were used in the first, third and fifth round, while plates coated with OVA and CIT-OVA were used in the rest. CIT-BSA and CIT-OVA were used alternately to avoid the scFvs reacted with the carrier proteins and to ensure the selection of scFvs specific to CIT [47].

To improve the affinity of the scFvs, other improvement strategies could be implemented in future experiments, such as the concentration of the coating proteins could be reduced gradually and the small molecules could be coated directly onto microtite plates [48]. In addition, the frequency and stringency of washing could be increased during the affinity selection to increase specificity.

### *E*. *coli* Expression of Anti-CIT ScFvs

About 140 colonies were sequenced and 32 clones were in the right reading frames with no stop codon. Prokaryotic expression of the selected scFvs was performed in *E*. *coli* BL21 (DE3) with the expression vector pTIG-TRX, which is known to increase the solubility of the expressed proteins. Periplasmic extracts and precipitates were collected after the cells were ruptured by sonication.

**Fig 8:**
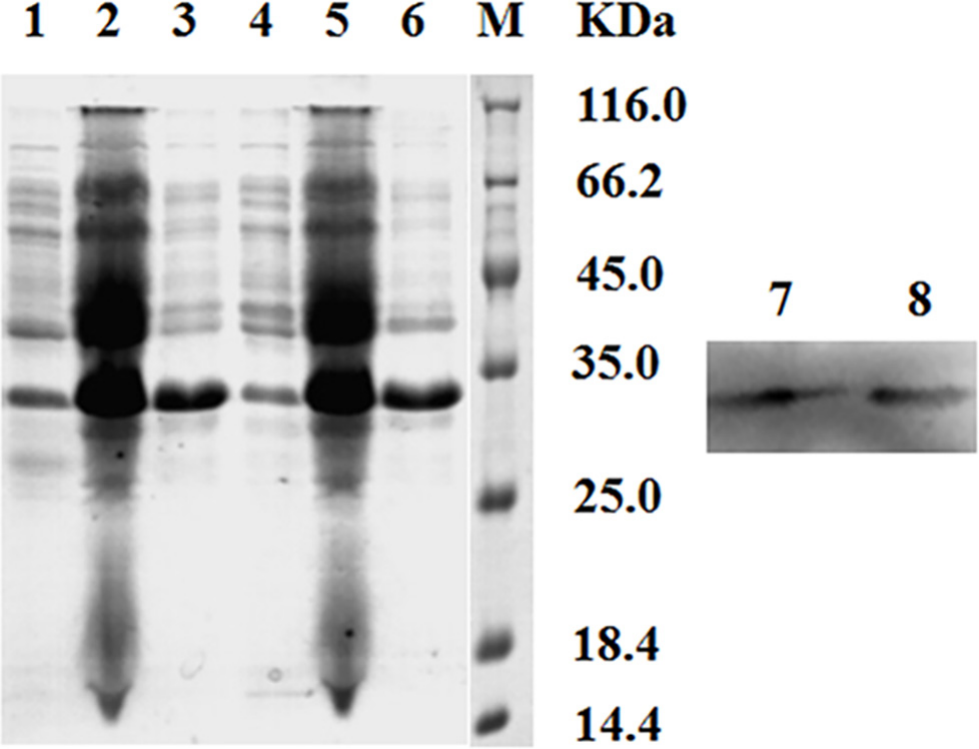
SDS-PAGE analysis and Western Blotting assay of two random chosen anti-CIT scFvs (about 32 kDa). Lane 1 and 4: SDS-PAGE analysis of the cellular supernatant after sonication; Lane 2 and 5: SDS-PAGE analysis of the whole cellular lysate after induction; Lane 3 and 6: SDS-PAGE analysis of the precipitate after sonication; Lane 7 and 8: Western Blotting assay of the cellular supernatant after sonication; Lane M: protein standards.

The SDS-PAGE analysis revealed that the expressed scFvs were found in both periplasmic extracts and precipitates, with a molecular weight of about 32 KDa (Fig 8). The concentration of IPTG and induction temperature should be optimized to prevent the formation of inclusion body during induction. Although the concentration of soluble scFvs in the supernatant was found to be lower than that in the inclusion bodies, the total amount of soluble scFvs in the supernatant was higher due to its larger volume.

Western blot of the periplasmic extracts showed specific detection of the soluble scFvs by the mouse anti-His tag antibody (Fig 8).

### Binding Activity of Selected Anti-CIT ScFvs

**Fig 9:**
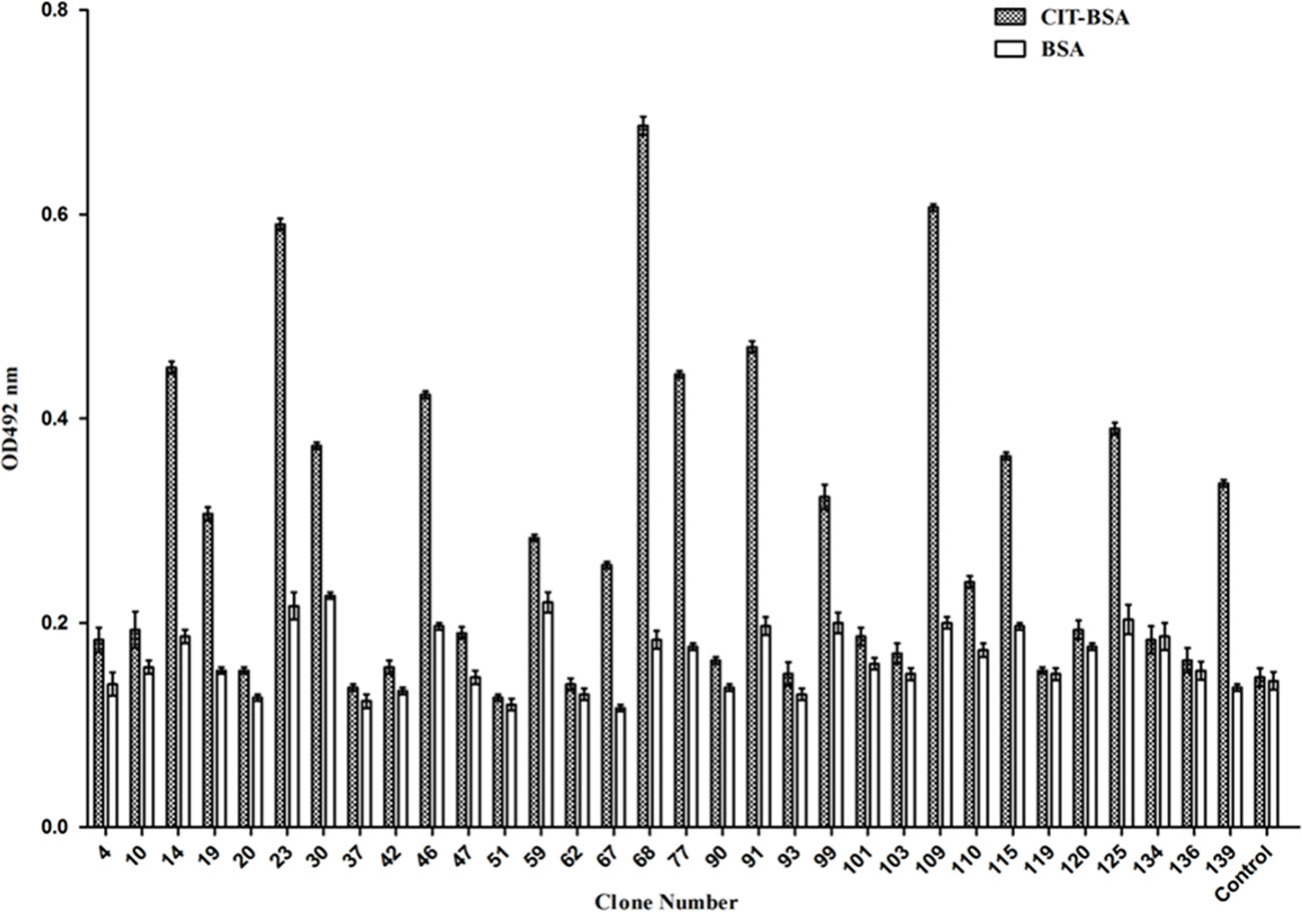
Indirect ELISA analysis of the binding activity of each clone to CIT-BSA and BSA (in triplicate). Control was the periplasmic extracts of the *Escherichia coli* BL21 (DE3) only with pTIG-TRX vector. The error bars represent the standard deviation.

The binding activity of the soluble scFvs was measured by indirect ELISA. A total of 13 clones showed binding activity with CIT-BSA, with clones 23, 68 and 109 showed the highest binding activity while no clone reacted with BSA (Fig 9). Given that other proteins expressed during induction could affect the indirect ELISA results, the absorbance values of the CIT-BSA could be higher while that of the control and BSA could be lower after the purification and concentration of the soluble scFvs.

### Specificity and Affinity Analysis of ScFvs

**Fig 10:**
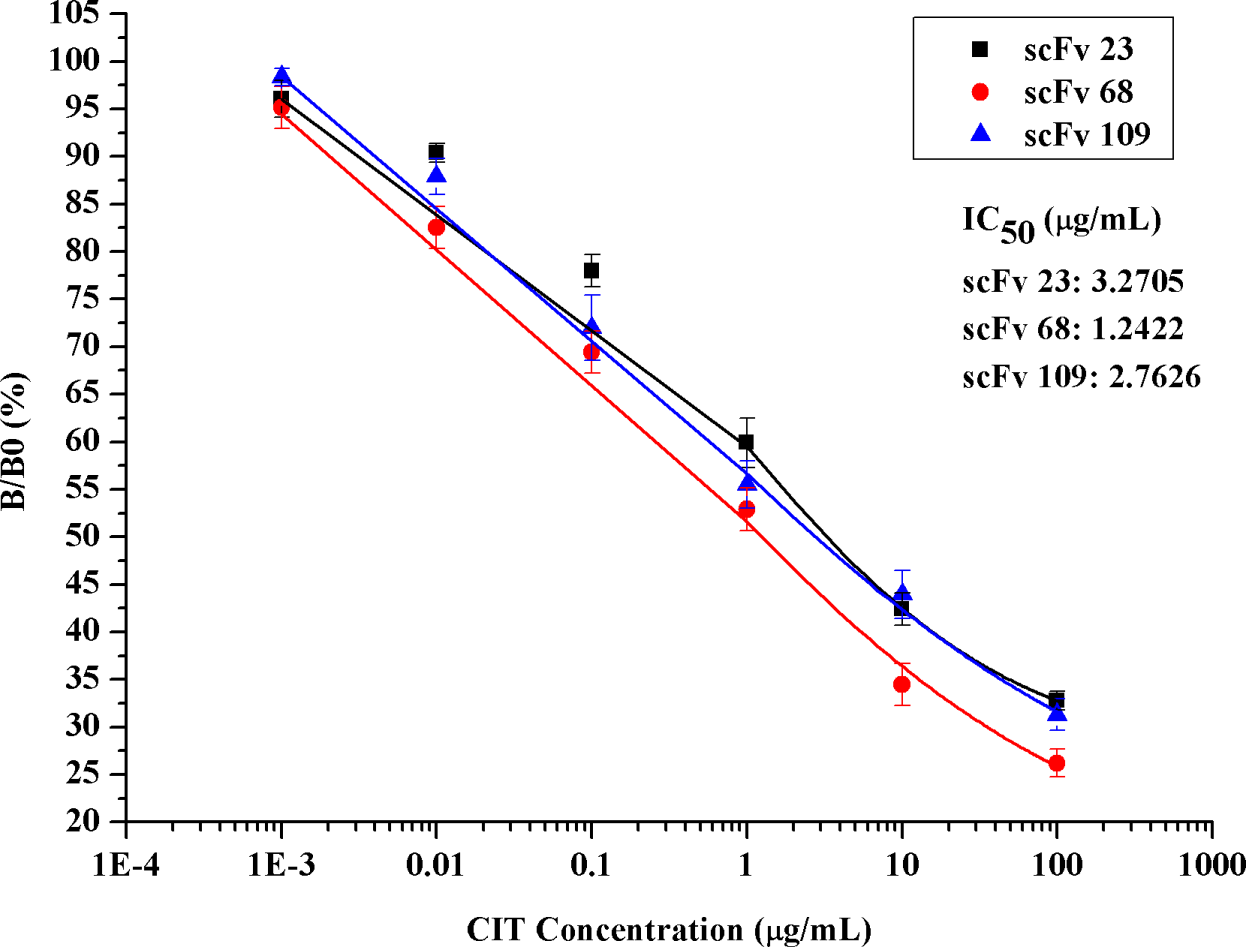
Indirect competitive ELISA analysis of the specificity and affinity of the scFvs 23, 68 and 109, respectively (in triplicate). Different concentrations of free CIT ranging from 0.001 μg/mL to 100 μg/mL were used to inhibit the binding of the scFvs to the CIT-BSA conjugate. B is the OD_492 nm_ at certain concentration of free CIT and B0 is the OD_492 nm_ at zero concentration of free CIT. The relative affinities of the scFvs were determined to be the reciprocal of molarity of the IC_50_ of CIT, which were calculated according to the four-parameter logistic equation. The error bars represent the standard deviation.

The specificity and affinity of the scFvs 23, 68 and 109 were measured by indirect competitive ELISA (Fig 10). The binding of each scFv to CIT-BSA was inhibited with different concentrations of free CIT, which indicated the scFvs were specific to CIT. There was a dose-dependent increase in inhibition and all the three scFvs exhibited specificity to CIT. The IC_50_ of the scFvs 23, 68 and 109 for CIT were 3.271, 1.242 and 2.763 μg/mL, while their limit of detection (LOD: IC_10_) were 0.011, 0.003 and 0.006 μg/mL, respectively. It could be concluded that the scFvs screened in this study were specific to CIT. The binding constants of the scFvs 23, 68 and 109 to CIT were determined to be 7.644 × 10^4^ M^-1^, 2.013 × 10^5^ M^-1^ and 9.049 × 10^4^ M^-1^, respectively. The scFvs showed higher binding activity towards CIT-BSA than that of the free CIT. This could be the following reasons [33, 49, 50]: 1, the non-immuned libraries were used; 2, the use of CIT-BSA as the ligand in the selection may favoure the enrichment of scFvs that preferentially bind the CIT-BSA; 3, the free CIT is not very soluble in aqueous solutions which may cause the concentration reduced; 4, some amino acid residues across BSA may contribute to the binding. To select scFvs with a higher affinity, affinity maturation of the scFvs by mutation and selection in vitro using ribosome display would be performed during the future research.

The cross-reactivity (CR) value was measured using the four mycotoxins, as shown in Table 1. The scFv 68 was unable to recognise any other mycotoxins except CIT and the CR value was less than 0.01%. It could be concluded that the scFv 68 was specific against CIT.

**Table 1:** Cross-reactivity of the scFv 68 with other mycotoxins.

Mycotoxins	PAT	AFB1	FB1	OTA	CIT
**Cross-reactivity (%)** ^**a**^	<10^−2^	<10^−2^	<10^−2^	<10^−2^	100

The reactivity to other mycotoxins was determined by indirect competitive ELISA and the values were calculated according to the four-parameter logistic equation.

^a^ Cross-reactivity (%) = (IC_50_ of CIT/IC_50_ of other mycotoxins) × 100.

**Fig 11:**
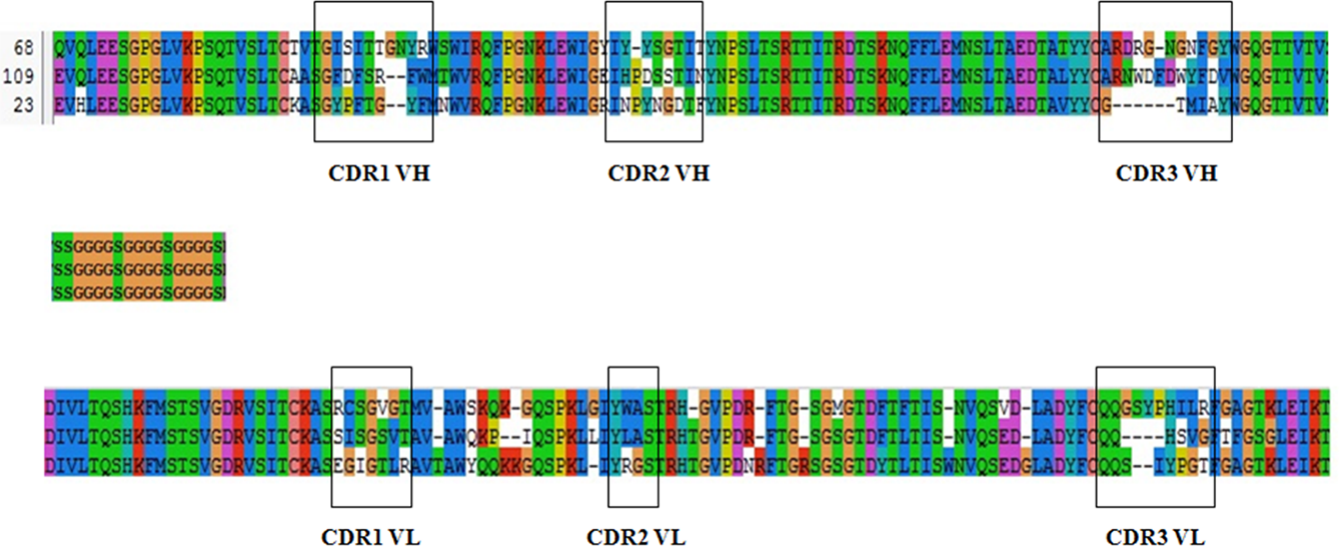
Alignment of the derived amino acids sequences of the three specific anti-CIT scFvs and complementary determining regions (CDRs). FRs and CDRs are determined by the V-BASE DNAPLOT software.

The derived amino acid sequences of the three specific anti-CIT scFvs and complementary determining regions (CDRs) were analyzed by the V-BASE DNAPLOT software (Fig 11). The heavy chains of scFvs 23, 68 and 109 belong to the V_H_1 gene family, V_H_3 gene family and V_H_4 gene family, respectively. All light chains belong to the IGKV19/28 subgroup. Although several amino acids residues of the CDRs were different, they shared a high level of homology with each other, where the V_H_ of scFv68 and scFv109 shared 85.7% homology and the V_L_ of the three scFvs shared 88.4% homology.

In this study specific anti-CIT scFvs have been selected and characterized from a scFv library by eukaryotic ribosome display technology. It is possible to use the selected scFvs to develop a novel immunoassay for CIT residues, while our work provides a reference for others to select scFvs specific to other small molecules using ribosome display. For future work, a low-fidelity DNA polymerase, DNA shuffling, error-prone PCR, directed evolution and crystallographic analysis could be used to evolve the scFvs to a wider affinity range [51, 52].
